# Astrocyte Function Is Affected by Aging and Not Alzheimer’s Disease: A Preliminary Investigation in Hippocampi of 3xTg-AD Mice

**DOI:** 10.3389/fphar.2019.00644

**Published:** 2019-06-06

**Authors:** Maria Rosanna Bronzuoli, Roberta Facchinetti, Marta Valenza, Tommaso Cassano, Luca Steardo, Caterina Scuderi

**Affiliations:** ^1^Department of Physiology and Pharmacology “V. Erspamer,” Sapienza University of Rome, Rome, Italy; ^2^Epitech Group SpA, Saccolongo, Italy; ^3^Department of Clinical and Experimental Medicine, University of Foggia, Foggia, Italy

**Keywords:** aging, Alzheimer’s disease, 3×Tg-AD mouse, astrocyte, connexin-43, AQP4, S100B, brain-derived neurotrophic factor

## Abstract

Old age is a risk factor for Alzheimer’s disease (AD), which is characterized by hippocampal impairment together with substantial changes in glial cell functions. Are these alterations due to the disease progression or are they a consequence of aging? To start addressing this issue, we studied the expression of specific astrocytic and microglial structural and functional proteins in a validated transgenic model of AD (3×Tg-AD). These mice develop both amyloid plaques and neurofibrillary tangles, and initial signs of the AD-like pathology have been documented as early as three months of age. We compared male 3×Tg-AD mice at 6 and 12 months of age with their wild-type age-matched counterparts. We also investigated neurons by examining the expression of both the microtubule-associated protein 2 (MAP2), a neuronal structural protein, and the brain-derived neurotrophic factor (BDNF). The latter is indeed a crucial indicator for synaptic plasticity and neurogenesis/neurodegeneration. Our results show that astrocytes are more susceptible to aging than microglia, regardless of mouse genotype. Moreover, we discovered significant age-dependent alterations in the expression of proteins responsible for astrocyte–astrocyte and astrocyte–neuron communication, as well as a significant age-dependent decline in BDNF expression. Our data promote further research on the unexplored role of astroglia in both physiological and pathological aging.

## Introduction

Alzheimer’s disease (AD) is currently considered a multifactorial disorder, although aging still remains its greatest risk factor (van der Flier and Scheltens, 2005; Hodson, 2018). Many targets are considered to design novel therapeutics. Glia represents one of them because of its contribution in the regulation of several highly specialized brain functions including glutamate, ions and water homeostasis, excitability and metabolic support of neurons, synaptic plasticity, brain blood flow, and neurotrophic support (Acosta et al., 2017; Bronzuoli et al., 2017). These functions are well integrated since astrocytes tightly communicate one to each other through gap junctions, comprised mainly of connexin-43 (CX43), that provide the structural basis for astrocyte networks (Bruzzone et al., 1996; Theis et al., 2005). These cells quickly respond to brain insults, synergistically working with microglia, the intrinsic immune effector of the brain, to remove injurious stimuli thus restoring brain homeostasis (Gehrmann et al., 1995; Scuderi et al., 2013). For example, during reactive astrogliosis, an event well described in both aged and AD brains (Scuderi et al., 2014a; Steardo et al., 2015; Rodríguez-Arellano et al., 2016), astrocytes modify their structure, usually studied by detecting the cytoskeletal glial fibrillary acidic protein (GFAP) and connexin expression (Peters et al., 2009; Giaume et al., 2010). Connexins form a “honeycomb” organization that creates edges between the end-feet enwrapping blood vessels. Here, astrocytes through aquaporin-4 (AQP4) water channels coordinate water flux and the clearance of interstitial fluid and neurotoxic solutes, including beta amyloid (Aβ). When such a clearance is dysfunctional, Aβ deposition occurs facilitating neurodegeneration (Iliff et al., 2012).

Astrocytes support neurons by releasing several neurotrophins, like S100B and the brain-derived neurotrophic factor (BDNF) (Marshak, 1990; Wiese et al., 2012). In the event of an abnormal production, as in some diseases including AD, such molecules contribute to neuronal damage (Vondran et al., 2010; Scuderi et al., 2014a; Bronzuoli et al., 2016). Glia involvement in the onset and/or progression of AD has been demonstrated by our and other groups (Esposito et al., 2007a; Esposito et al., 2007b; Esposito et al., 2011; Scuderi et al., 2011; Scuderi et al., 2012; Scuderi et al., 2014b; Scuderi and Steardo, 2013; Cipriano et al., 2015; Salter and Stevens, 2017; Taipa et al., 2017). Less is known about their role during healthy aging. Therefore, in this brief research report, we provide a preliminary descriptive investigation of the effects of aging on morphology and functions of hippocampal astrocytes and microglia by comparing young adult (6-month-old) and aged (12-month-old) healthy (Non-Tg) and AD-like (3×Tg-AD) mice to model healthy and pathological aging, respectively. We explored the hippocampal expression of GFAP and S100B for astrocytes, and the ionized calcium binding adaptor molecule-1 (Iba1) and the cluster of differentiation 11b/c (CD11b/c) for microglia. Moreover, we examined deeper astrocyte functions by exploring AQP4 and CX43 expression. Since one of the most important astrocytes functions is the neurotrophic support (Kimelberg and Nedergaard, 2010), we also investigated BDNF production and the dendritic microtubule-associated protein 2 (MAP2).

Collectively, our findings reveal that aging negatively alters astrocytic functions, including their neurotrophic support. A main effect of aging and not of genotype was detected in all astrocytic markers here studied, thus suggesting that observed alterations to astroglia functions were related to aging itself rather than AD.

## Materials and Methods

All procedures involving animals were approved by the Italian Ministry of Health (Rome, Italy) and performed in compliance with the guidelines of the Directive 2010/63/EU of the European Parliament, and the D.L. 26/2014 of Italian Ministry of Health.

### Animals

Six- and 12-month-old male 3×Tg-AD mice (homozygous for PS1_M146V_ and homozygous for the co-injected APP_swe_, tau_P301L_ transgenes) were used as model of pathological aging. This mutant mouse exhibits, indeed, AD-like plaques and tangles associated with synaptic dysfunction (Oddo et al., 2003a), observed also in our experimental conditions (see **Supplementary Figure S1**). To reproduce a condition of healthy aging, age-matched wild-type littermates (Non-Tg) (C57BL6/129SvJ) were used. Mice were housed in an enriched environment at controlled conditions (22 ± 2°C temperature, 12-h light/12-h dark cycle, 50–60% humidity), with *ad libitum* food and water. Six male mice per group were decapitated, and their brains rapidly isolated and either flash frozen in 2-methylbutane to perform immunofluorescences or dissected to isolate hippocampi for western blot analyses. Tissues were stored at −80°C.

### Western Blot

Hippocampi were processed as previously described (Scuderi et al., 2018a). Briefly, tissues were homogenized in ice-cold hypotonic lysis buffer and then centrifuged. Fifty micrograms of proteins was resolved on 12% acrylamide SDS-PAGE gels and then transferred onto nitrocellulose membranes, which were blocked for 1 h with either 5% bovine serum albumin (BSA) (Fitzgerald, MA) or non-fat dry milk (Bio-Rad, Italy) in tris-buffered saline-0.1% tween-20 (Corning, NY). Membranes were then incubated overnight with one of the following primary antibodies: rabbit anti-GFAP (1:25,000; Abcam, UK), rabbit anti-S100B (1:1,000; Novus Biological, CO), rabbit anti-Iba1 (1:1,000; Abcam), rabbit anti-CD11b/c (1:1,000; Bioss, MA), mouse anti-CX43 (1:500; EMD Millipore, MA), mouse anti-AQP4 (1:500; Santa Cruz, TX), rabbit anti-BDNF (1:1,000; Abcam), mouse anti-β-amyloid (1:200; Millipore, Germany), rabbit anti-p[Ser396]tau (1:1,000; Thermo Fisher Scientific, MA). Rabbit anti-β-actin (1:1,500, Santa Cruz) was used as loading control. After rinses, membranes were incubated with the proper secondary horseradish peroxidase (HRP)-conjugated antibody (1:10,000–1:30,000; Jackson ImmunoResearch, UK), and immunocomplexes detected by an ECL kit (GE Healthcare Life Sciences, Italy). Signals were analyzed by ImageJ.

### Immunofluorescence

As previously described (Bronzuoli et al., 2018), hippocampal coronal slices (12-μm thickness) obtained at a cryostat were post-fixed with 4% paraformaldehyde (Sigma-Aldrich). After blockage in 1% BSA dissolved in PBS/0.25% triton X-100, slices were incubated overnight with one of the following primary antibodies: mouse anti-CX43 (1:50, EMD Millipore), mouse anti-AQP4 (1:50, Santa Cruz), rabbit anti-GFAP (1:1000, Abcam), mouse anti-MAP2 (1:250, Novus Biologicals). Sections were rinsed in PBS and incubated for 2 h with the proper secondary antibody [1:200 fluorescine-affinipure goat anti-rabbit IgG (H+L); 1:300-1:400 rhodamine-affinipure goat anti-mouse IgG (H+L) (Jackson ImmunoResearch)] and DAPI (1:75,000, Sigma-Aldrich). Fluorescence was detected by an Eclipse E600 microscope (Nikon, Japan). To avoid the observation of differences among groups caused by artifacts, the exposure parameters, including gain and time, were kept uniform during image acquisitions. Pictures were captured by a QImaging camera and analyzed by ImageJ. Immunofluorescence quantifications are expressed as ΔF/F_0_ = [(F− F_0_)/F_0_], where F is the mean fluorescence intensity and F_0_ is the mean background fluorescence. We performed immunofluorescence experiments in the hippocampus, focusing our analyses on the Ammon’s horn 1 (CA1) subregion because this area is one of the most vulnerable to AD, in both patients (Rössler et al., 2002; Mueller et al., 2010) and 3×Tg-AD mice (Oddo et al., 2003a). We analyzed three serial coronal sections per animal (between −1.82 and −1.94 mm from bregma) spaced 36 µm apart, analyzing four ROIs in the stratum radiatum of each section (200 × 100 µm).

### Statistics

Data were analyzed by two-way ANOVA using GraphPad Prism6. When applicable, Bonferroni’s *post hoc* test was used. Data were expressed as mean ± standard error of the mean (SEM) of percentage of control (6-month-old/Non-Tg mice).

## Results

### Aging Affects Morphology and Functions of Hippocampal Astrocytes, Independent of Genotype

**Figure 1:**
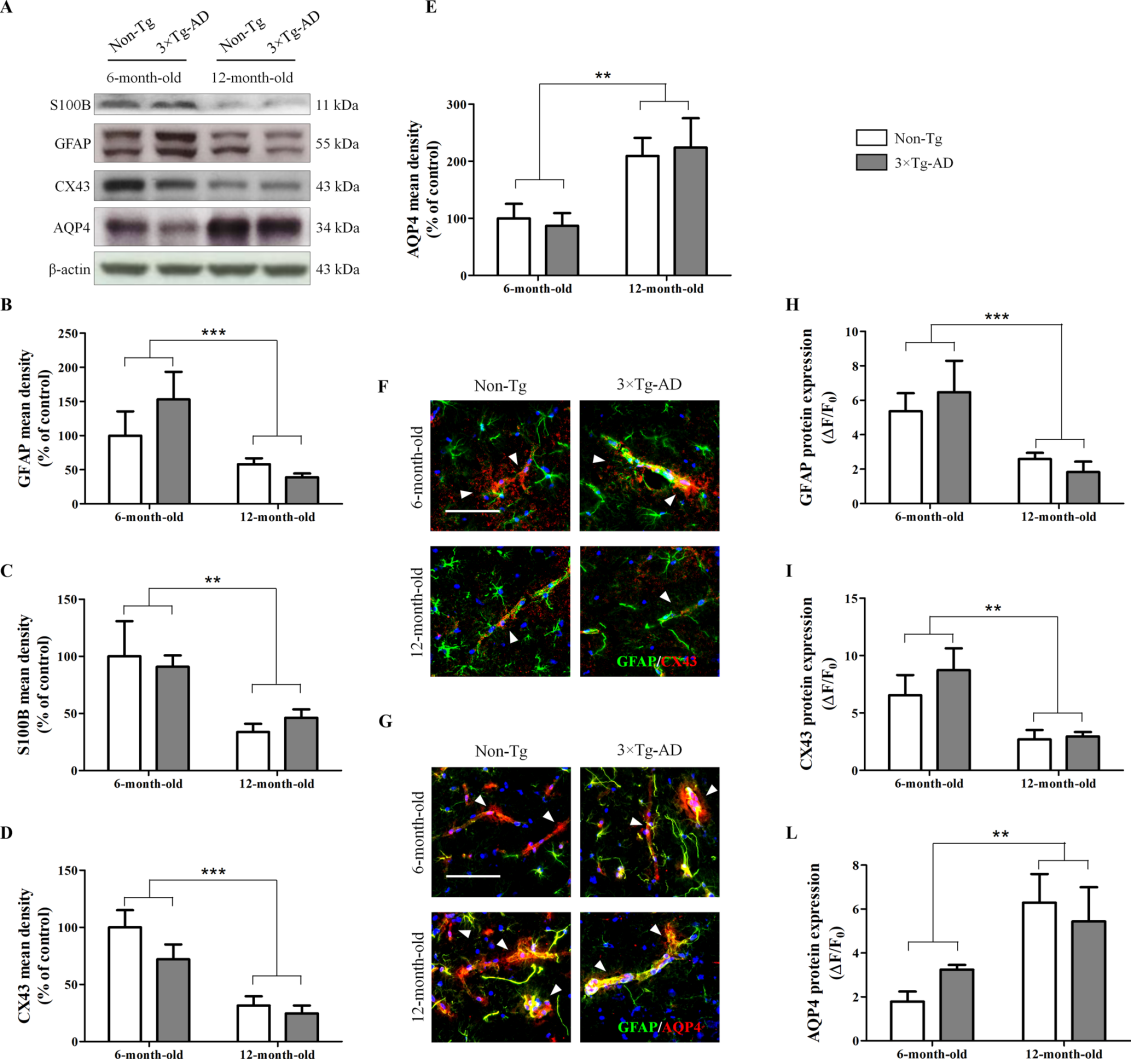
Effects of aging on morphology and functions of hippocampal astrocytes. **(A)** Representative western blots for GFAP, S100B, CX43 and AQP4 and **(B**, **C**, **D**, **E)** densitometric analyses normalized to β-actin loading control. Results are expressed as means ± SEM of percentage of controls (6-month-old/Non-Tg) (N = 3, in triplicate). **(F)** Representative fluorescent photomicrographs of CX43 (red) and GFAP (green) in the hippocampal CA1 region of both 6- and 12-month-old Non-Tg and 3×Tg-AD mice. White arrows indicate CX43 mainly expressed in astrocytes enveloping blood vessels. **(G)** Representative fluorescent photomicrographs of AQP4 (red) and GFAP (green) in the hippocampal CA1 region of both 6- and 12-month-old Non-Tg and 3×Tg-AD mice. White arrows indicate AQP4 expressed in astrocyte end-feet surrounding blood vessels. Fluorescence analyses of **(H)** GFAP, **(I)** CX43 and **(L)** AQP4 are expressed as ΔF/F_0_ = [(F− F_0_)/F_0_], where F is the mean fluorescence intensity and F_0_ is the mean background fluorescence. Nuclei were stained with DAPI (blue). Scale bar 50 µm. Statistical analysis was performed by two-way ANOVA followed by Bonferroni’s *post hoc* test (**p < 0.01; ***p < 0.001, 6-month-old vs 12-month-old).

Results from Western blot experiments, performed in homogenates of hippocampi of Non-Tg and 3×Tg AD mice, showed that age significantly affects astrocyte morphology and functions. In fact, we observed a significant reduction of the cytoskeletal protein GFAP and the neurotrophin S100B in 12-month-old mice compared with 6-month-old mice, irrespective of genotype (**Figure 1A, B, C**). Moreover, we found a significant genotype-by-age interaction on GFAP data (*p* = 0.0357). By immunofluorescence, we observed a significant reduction of GFAP in the hippocampal CA1 subregion of 12-month-old mice compared with 6-month-old mice, independently of genotype (**Figure 1F, G, H**). In addition, results from immunofluorescence and Western blot revealed that aging impacts on astrocyte functions. Indeed, we found a significant decrease of CX43 expression in 12-month-old mice compared with 6-month-old mice, independently of genotype (**Figure 1A, D, F, I**). Moreover, in the same experimental conditions, we observed an increased expression of AQP4 in the hippocampi of aged mice regardless of genotype (**Figure 1A, E, G, L**). For both AQP4 and CX43, no genotype-by-age interaction was detected.

### Aging Does Not Significantly Affect Hippocampal Microglia

**Figure 2:**
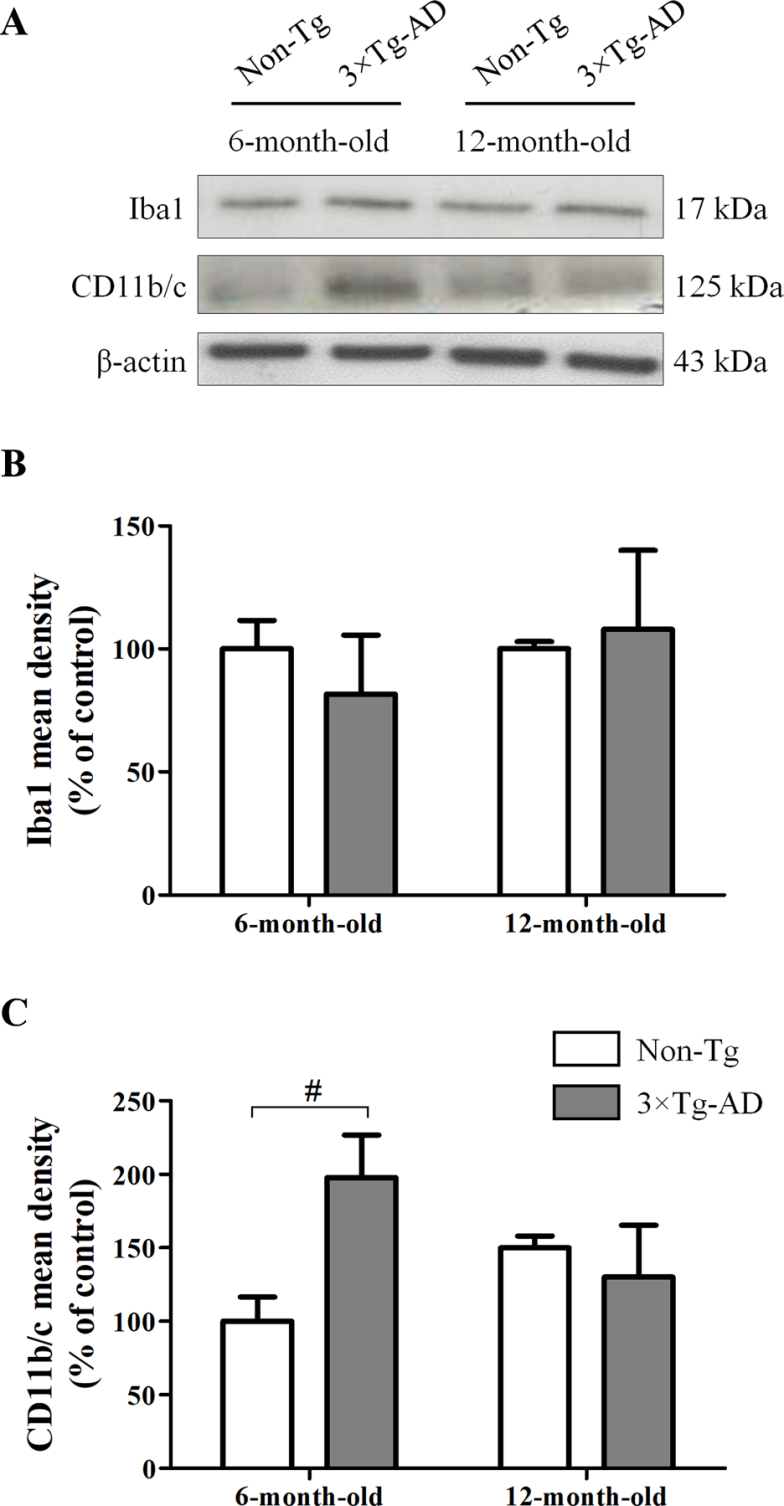
Effects of aging on hippocampal microglia of 3×Tg-AD and Non-Tg mice. **(A)** Representative Western blots for Iba1 and CD11b/c and **(B**, **C)** densitometric analyses normalized to β-actin loading control. Results are expressed as means ± SEM of percentage of controls (6-month-old/Non-Tg) (N = 3, in triplicate). Statistical analysis was performed by two-way ANOVA followed by Bonferroni’s *post hoc* test (^#^ *p* < 0.05, 6-month-old/3×Tg-AD vs 6-month-old/Non-Tg).

In hippocampal homogenates of Non-Tg and 3×Tg-AD mice, we performed Western blot experiments for Iba1, a calcium-binding protein constitutively expressed by both surveillant and activated microglia, and CD11b/c, a marker of proliferative reactivity. Obtained results showed that the expression of Iba1 was not affected by either age or genotype (**Figure 2A, B**). However, we observed a significant increase of CD11b/c in 6-month-old 3×Tg-AD mice in comparison with their age-matched Non-Tg littermates, indicative of a potential microglial activation in young transgenic animals, but not in aged ones (**Figure 2A, C**).

### Aging Reduces Hippocampal Expression of BDNF, With No Significant Impact on Neuronal Loss, Independent of Genotype

**Figure 3:**
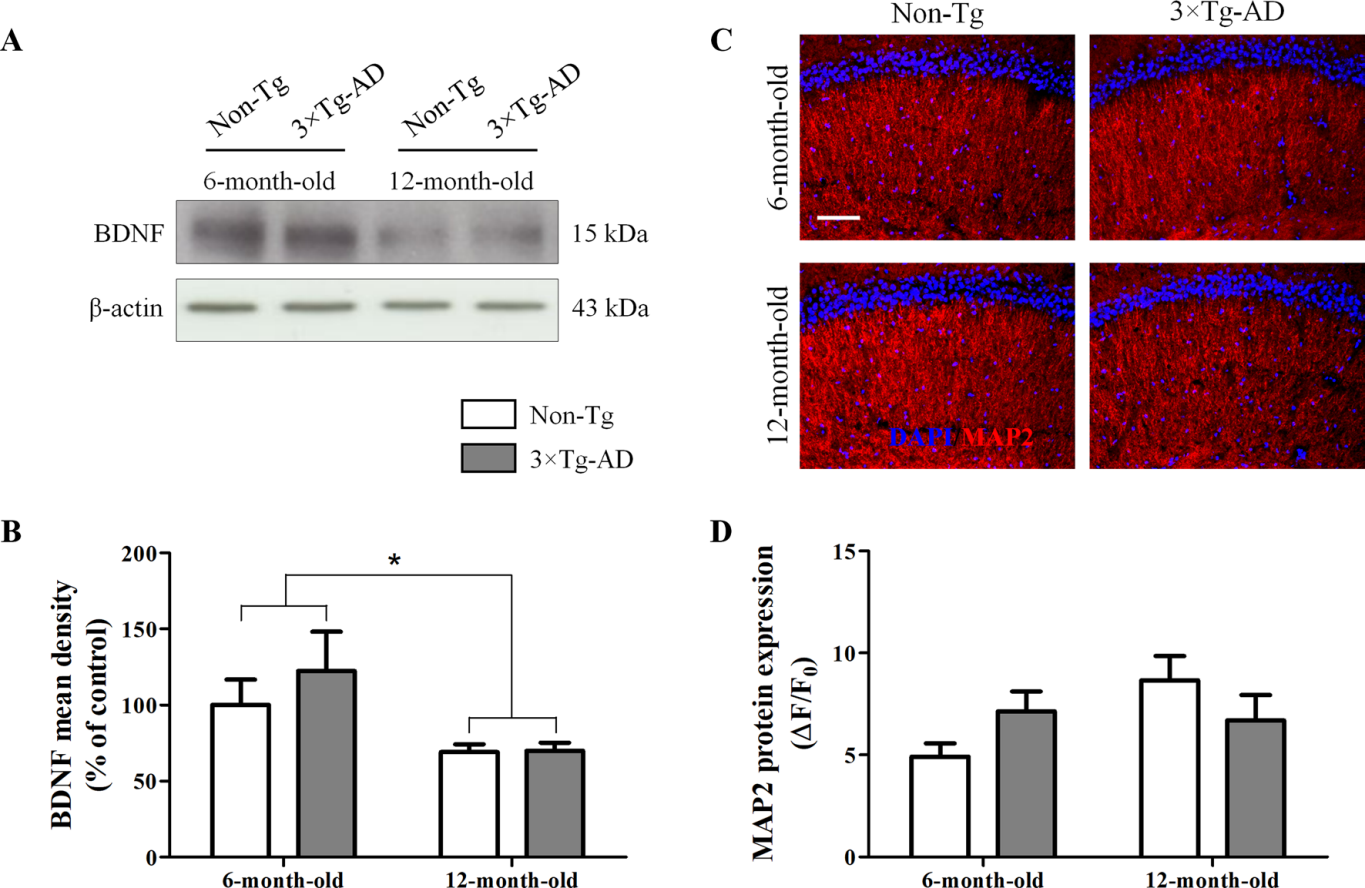
Effects of aging on BDNF and MAP2 expression in hippocampus of 3×Tg-AD and Non-Tg mice. **(A)** Representative Western blots for BDNF and **(B)** densitometric analysis normalized to β-actin as loading control. Results are expressed as means ± SEM of percentage of controls (6-month-old/Non-Tg) (N = 3, in triplicate). **(C)** Representative fluorescent photomicrographs of MAP2 (red) in the hippocampal CA1 region of both 6- and 12-month-old Non-Tg and 3×Tg-AD mice. **(D)** MAP2 fluorescence analysis is expressed as ΔF/F_0_ = [(F− F_0_)/F_0_], where F is the mean fluorescence intensity and F_0_ is the mean background fluorescence. Nuclei were stained with DAPI (blue). Scale bar 50 µm. Statistical analysis was performed by two-way ANOVA followed by Bonferroni’s *post hoc* test (*p < 0.05; 6-month-old vs 12-month-old).

The neurotrophic factor BDNF is produced by neurons and, only under pathological circumstances, by astrocytes (Parpura and Zorec, 2010; Fulmer et al., 2014). Therefore, we tested whether aging could affect BDNF production and, in turn, cause neuronal loss. Results from Western blot experiments, performed in homogenates of hippocampi of Non-Tg and 3×Tg AD mice, showed a significant age-related decrease in BDNF production, irrespective of genotype (**Figure 3A, B**). Despite the observed reduction of this important neurotrophic factor, the hippocampal expression of the dendritic marker MAP2, analyzed by immunofluorescence, was not significantly different between all experimental groups (**Figure 3C, D**).

## Discussion

In the present study, we provide the first preliminary evidence of the effect of aging on structure and functions of hippocampal glial cells. Our primary goal was to study the impact of age on glial cells in conditions of physiological and pathological, AD-like, aging. To start addressing this issue, we used young adult and aged Non-Tg and 3×Tg-AD mice to simulate healthy and pathological aging, respectively. Collectively, our results indicate that aging affects astrocytes with no significant differences between the two genotypes.

The importance of glia in maintaining brain homeostasis and cerebral metabolism is well documented (Parpura and Haydon, 2008; Dzamba et al., 2016). Growing evidence demonstrate the fundamental role of these cells in the etiopathogenesis of several neuropsychiatric disorders thus opening new scenarios to the development of glia-targeted drugs (Bronzuoli et al., 2017; Bronzuoli et al., 2018; Bronzuoli et al., 2019; Cartocci et al., 2018; Scuderi et al., 2018a). The role of glia in healthy aging is still poorly investigated. No data are yet available elucidating whether glial abnormalities involved in neurodegeneration were due to disease progression or they were just a consequence of aging itself. Therefore, we compared young adult (6-month-old) and aged (12-month-old) healthy (Non-Tg) and AD-like (3×Tg-AD) mice. 3×Tg-AD mice progressively and hierarchically develop Aβ plaques and neurofibrillary tangles in AD-relevant brain regions (cortex, hippocampus, and amygdala) and show an age-related cognitive decline that closely mimics the human AD progression (Oddo et al., 2003b; Cassano et al., 2011; Cassano et al., 2012; Romano et al., 2014; Coughlan et al., 2018). Here, we demonstrate that aging affects glial cells, especially modifying astrocyte structure and functions. In our experimental conditions, we observed that aging reduces the expression of the cytoskeletal GFAP and the neurotrophin S100B, regardless of mice genotype. The age-dependent increase in astrocyte reactivity has been well documented (Beach et al., 1989; David et al., 1997; Janota et al., 2015). However, data obtained from both human material and animal models demonstrate the existence of a complex and region-specific glial response in AD and aging that can be crudely summarized in glial reactivity or glial degeneration, atrophy, and loss of functions (Rodríguez et al., 2016; Verkhratsky et al., 2016; Scuderi et al., 2018b). Our findings are apparently in contrast with the evidence indicating an age-matched astrogliosis in 3×Tg-AD mice (Oddo et al., 2003a; Zaheer et al., 2013). However, Oddo and colleagues analyzed cortical and hippocampal levels of GFAP in both 3×Tg-AD and Non-Tg mice showing no substantial difference between genotype in the hippocampal levels of GFAP in agreement with the results of the present paper (Oddo et al., 2003a). GFAP expression showed a trend toward being upregulated with increasing age in their experimental conditions; however, they compared 2-month-old mice with 16-month-old mice. The different age chosen for comparison could explain the discrepancy with the present data. 3×Tg-AD mice show activated astrocytes and microglia as age increases and the majority of the studies detected them near amyloid plaques (Oddo et al., 2003a; Zaheer et al., 2013; Rodríguez-Arellano et al., 2016). Interestingly, numerous investigators reported the concomitant occurrence of astrogliosis and astroglial atrophy, demonstrating that the latter appears as a generalized process, whereas astrogliosis is triggered by senile plaques and Aβ aggregates (Rodríguez et al., 2009; Olabarria et al., 2010; Heneka et al., 2010; Verkhratsky et al., 2010; Yeh et al., 2011). In parallel with activation, the AD progression is also associated with astrodegeneration. Accordingly, atrophic astrocytes have been observed in the hippocampus, prefrontal and entorhinal cortices of mouse models of AD (Verkhratsky et al., 2016). In line with these observations, our group has recently demonstrated a reduction of GFAP in the hippocampus of aged 3×Tg-AD mice (Scuderi et al., 2018a). Interestingly, Hoozemans and colleagues (2011) showed lower GFAP immunostaining in post-mortem brains from old AD patients (>80 years) compared with younger AD cases, concluding that the occurrence of astrocyte activation decreases with increasing age in AD dementia.

In our experimental conditions, aging negatively affected astrocyte functions, with particular regard to some of their homeostatic and neurotrophic roles. We found, in both 3×Tg-AD and Non-Tg mice, a significant reduction of CX43 expression, one of the major gap junction proteins that allow an efficient communication among astrocytes. Our data are in line with those obtained by Cotrina and collaborators (2001) demonstrating an age-dependent CX43 reduction in C57Bl/6 mice. The impairments observed in our experimental conditions seems to be caused by aging; in fact, we did not observe any significant differences between the two genotypes. We speculate that this finding together with the aforementioned astrocyte atrophy could be due to a reduced communication among astrocytes. This communication is usually mediated by endogenous peptides, growth factors, neurotransmitters, bioactive lipids, and structural proteins widely expressed in the astrocytic end-feet enveloping blood vessels (Rouach et al., 2002). Interestingly, some authors demonstrated that CX43 reduction boosts Aβ deposits (Koulakoff et al., 2012). In this context, our data could suggest a mechanism responsible for the observed deposition of a huge amount of Aβ in the brain of healthy subjects not affected by AD (Chételat et al., 2013).

AQP4 is another astrocytic protein implicated in the CNS lymphatic drainage and clearance of interstitial solutes, including Aβ (Cotrina et al., 2001; Iliff et al., 2012; Yang et al., 2016). We found that aging is responsible for the augmented AQP4 expression, independent of genotype. Accordingly, AQP4 is highly expressed near Aβ plaques in amyloidopathies (Moftakhar et al., 2010). The raise in AQP4 expression could be a compensatory process aimed at keeping water and ion homeostasis, and at encouraging the clearance of the interstitial fluid in the CNS in both physiological and pathological aging (Gupta and Kanungo, 2013). Interestingly, our data on CX43 and AQP4 are in line with those we obtained on Aβ_(1-42)_ levels (**Supplementary Figure S1**). Indeed, we found higher hippocampal Aβ_(1-42)_ levels in aged mice of both genotypes. We also observed a significant increase of Aβ_(1-42)_ expression in 3×Tg-AD mice in comparison to Non-Tg animals, at both ages. Given these results, further experiments are required to look for a correlation between severity of pathology and astrocyte protein expression, also investigating other areas crucially involved in AD.

Quite unexpectedly, we did not observe significant structural modifications in microglia in our experimental conditions, except for a significant activation in young transgenic mice, supposedly indicative of the presence of an early pro-inflammatory status which disappears in adult mice. These findings are in line with those previously obtained in 3×Tg-AD mice showing that neuroinflammation occurs early in AD-like pathology (Bronzuoli et al., 2018; Scuderi et al., 2018a), and with some authors suggesting that neuroinflammation becomes less and less evident and spread as the disease progresses, remaining detectable in the proximity of Aβ plaques and neurofibrillary tangles (Yeh et al., 2011; Rodríguez-Arellano et al., 2016; Verkhratsky et al., 2016).

The role of microglia in aging and AD is complex and not fully elucidated. Microglial pro-inflammatory markers, including fractalkine and receptors, are reduced in the brain of aged mice compared to adult controls (Wynne et al., 2010), and microglia in aged animals appear irregularly distributed, with variable morphology of both cell bodies and processes, occupying smaller territories (Tremblay et al., 2012). Moreover, it has been demonstrated that 3×Tg-AD mice at 2, 3, and 6 months progressively show significant microglia activation in the entorhinal cortex but not in the hippocampus (Janelsins et al., 2005), as well as a significant increase of activated microglia at 18 months of age compared to 3×Tg-AD mice at 9 months, but not at 12 months of age (Rodríguez et al., 2013). Conversely, very recently Belfiore and colleagues (2019) have demonstrate an age-dependent activation of hippocampal microglia in female 3×Tg‐AD mice, from 6 up to 20 months of age. The heterogeneity of these results confirms that categorizing microgliosis is still particularly challenging. Nowadays, several authors hypothesize the presence of diverse microglial reactions to different disease stages, suggesting that the complete characterization of these processes may open new avenues for therapeutic intervention (Mosher and Wyss-Coray, 2014; Sarlus and Heneka, 2017). Based on these considerations and keeping in mind that our study is preliminary, we believe that further experiments using more specific markers will be required to better characterize the microglia phenotype and its correlations with the neuroinflammatory process also investigating other brain areas importantly affected by AD.

Astrocytes produce and release several neurotrophins, including S100B and BDNF. S100B is a calcium-binding protein mainly involved in cell cycle progression and differentiation as well as neurite outgrowth (Scotto et al., 1998; Sen and Belli, 2007). BDNF is produced mainly by astrocytes over neurons under pathological conditions (Dougherty et al., 2000). We found a significant reduction of both BDNF and S100B in aged mice, irrespective of genotype. Since BDNF regulates the astrocytic expression of S100B and both together are required to support at least serotonergic neurons (Ye et al., 2011), further studies would elucidate the cross-talk between astrocytes and neuronal cells. Despite the reduced neurotrophic support, we did not detect neuronal impairment, as assessed by MAP2.

In conclusion, in this brief research report, we provide preliminary data demonstrating that aging rather than AD progression importantly affects morphology and functions of hippocampal glial cells. In our experimental conditions, the most vulnerable cells were the astrocytes whose structure and functions appear profoundly modified. Additional studies are required to further reveal the role of astrocytes and microglia in both physiological and pathological aging, using more specific markers for the detection of changes in their morphology and/or functions, and extending such observations in other brain regions. Avoiding any superficial projection to human disease and keeping in mind that human astrocytes are more complex than their murine counterparts, these data open novel perspective in the field of astrocyte functions in health and disease.

## Data Availability Statement

Datasets are available on request. Requests to access the datasets should be directed to caterina.scuderi@uniroma1.it.

## Ethics Statement

All procedures involving animals were approved by the Italian Ministry of Health (Rome, Italy) and performed in compliance with the guidelines of the Directive 2010/63/EU of the European Parliament, and the D.L. 26/2014 of Italian Ministry of Health.

## Author Contributions

MB, RF, MV, and CS performed most of the molecular experiments and analyzed the data. TC housed and raised the animals. LS, TC, and CS supervised the experiments and discussed the results. MB, RF, MV, and CS wrote the manuscript. All authors contributed to and approved the final manuscript.

## Funding

This work was supported by the Italian Ministry of Instruction, University and Research (MIUR) to LS (PON01-02512, PRIN prot. 2009NKZCNX, PRIN prot. 2015HRE757) and to CS (PRIN prot. 2015KP7T2Y_002); the SAPIENZA University of Rome to CS (prot. C26A15X58E and prot. MA116154CD981DAE) and MB (prot. C26N15BHZZ).

## Conflict of Interest Statement

MV is currently employed by company Epitech Group SpA, which had no role in study design, collection, analysis, and interpretation of data, in the writing of the report, and in the decision to submit the paper for publication. The remaining authors declare that the research was conducted in the absence of any commercial or financial relationships that could be construed as a potential conflict of interest.

## Supplementary Material

Figure S1 Effects of aging on Aβ_(1-42)_ production and tau protein phosphorylation in hippocampus of 3×Tg-AD and Non-Tg mice. **(A)** Representative Western blots for Aβ_(1-42)_ and p[Ser396]tau and **(B**, **C)** densitometric analyses normalized to β-actin as loading control. Results are expressed as means ± SEM of percentage of controls (6-month-old/Non-Tg) (N = 3, in triplicate). Statistical analysis was performed by two-way ANOVA followed by Bonferroni’s *post hoc* test (***p*< 0.01; ****p*< 0.001, 6-month-old vs 12-month-old; ^##^ *p*< 0.01, 6-month-old/3×Tg-AD vs 6-month-old/Non-Tg; ^###^ *p*< 0.001, 12-month-old/3×Tg-AD vs 12-month-old/Non-Tg).
