# Supplementary figures and images for: Astrocyte Function Is Affected by Aging and Not Alzheimer’s Disease: A Preliminary Investigation in Hippocampi of 3xTg-AD Mice
Source: Front Pharmacol. 2019 Jun 6;10:644. doi: 10.3389/fphar.2019.00644 (PMC6562169; doi:10.3389/fphar.2019.00644)

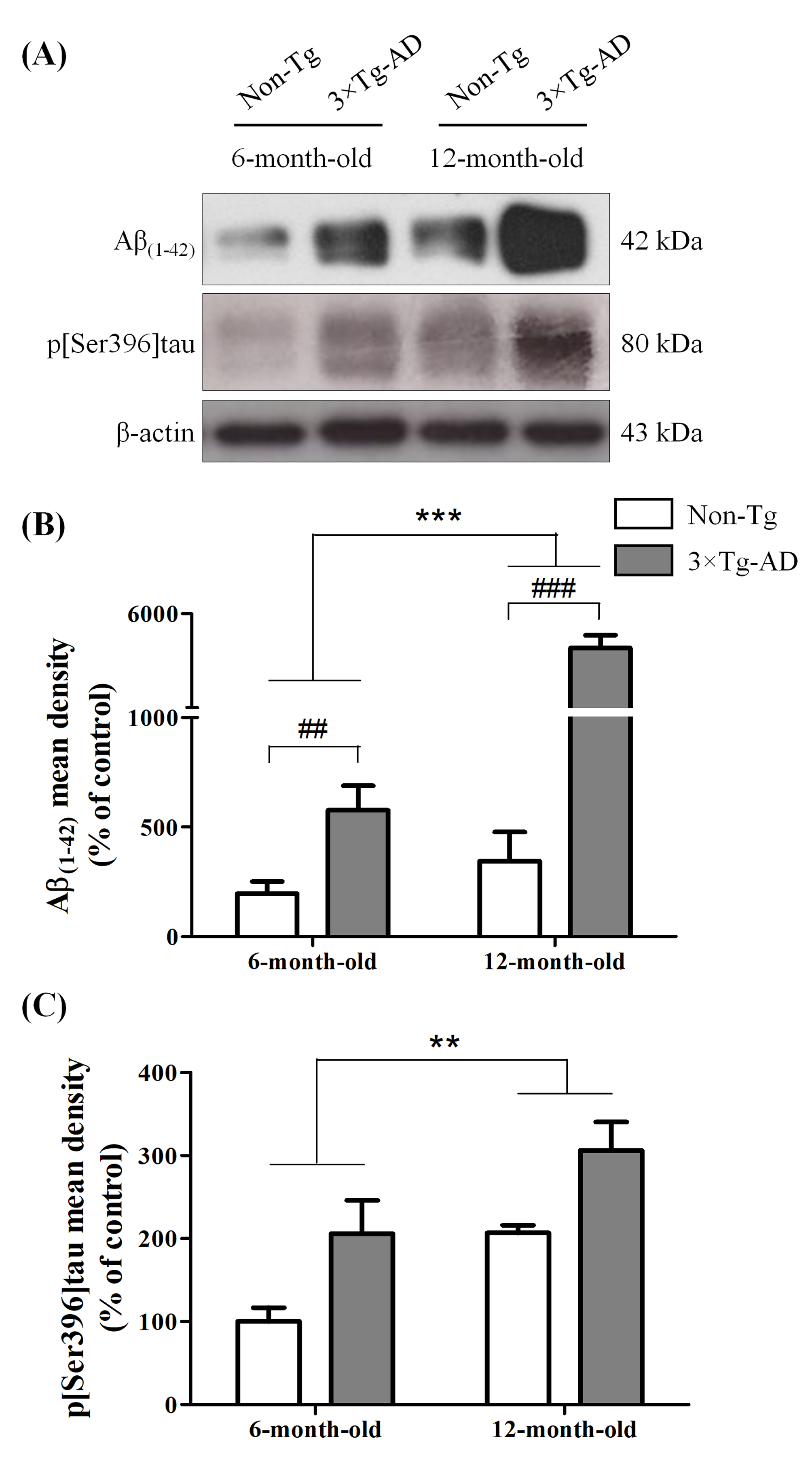

Supplement: Figure S1 — Effects of aging on Aβ(1-42) production and tau protein phosphorylation in hippocampus of 3×Tg-AD and Non-Tg mice. (A) Representative Western blots for Aβ(1-42) and p[Ser396]tau and (B, C) densitometric analyses normalized to β-actin as loading control. Results are expressed as means ± SEM of percentage of controls (6-month-old/Non-Tg) (N = 3, in triplicate). Statistical analysis was performed by two-way ANOVA followed by Bonferroni’s post hoc test (**p< 0.01; ***p< 0.001, 6-month-old vs 12-month-old; ## p< 0.01, 6-month-old/3×Tg-AD vs 6-month-old/Non-Tg; ### p< 0.001, 12-month-old/3×Tg-AD vs 12-month-old/Non-Tg). [file Image_1.tif]
